# An Open-Source Joystick Platform for Investigating Forelimb Motor Control, Auditory-Motor Integration, and Value-Based Decision-Making in Head-Fixed Mice

**DOI:** 10.1523/ENEURO.0038-25.2025

**Published:** 2025-04-18

**Authors:** Ivan Linares-García, Evan A. Iliakis, Sofia E. Juliani, Alexandra N. Ramirez, Joel Woolley, Edgar Díaz-Hernández, Marc V. Fuccillo, David J. Margolis

**Affiliations:** ^1^Department of Cell Biology and Neuroscience, Rutgers, The State University of New Jersey, Piscataway, New Jersey 08854; ^2^Department of Neuroscience, Perelman School of Medicine, University of Pennsylvania, Philadelphia, Pennsylvania 19104

**Keywords:** auditory-motor, joystick, learning, mouse behavior, open-source, value-based

## Abstract

Investigation of neural processes underlying motor control requires behavioral readouts that capture the richness of actions, including both categorical (choice-based) information and motor execution (kinematics). We present an open-source platform for behavioral training of head-fixed mice that combines a stationary or retractable forelimb-based joystick, sound-presentation system, capacitive lick sensor, and water reward dispenser. The setup allows for the creation of multiple behavioral paradigms, two of which are highlighted here: a two-alternative forced-choice auditory-motor discrimination paradigm and a two-armed bandit value-based decision-making task. In the auditory-motor paradigm, mice learn to report high- or low-frequency tones by pushing or pulling the joystick. In the value-based paradigm, mice learn to push or pull the joystick based on the history of rewarded trials. In addition to reporting categorical choices, this setup provides a rich dataset of motor parameters that reflect components of the underlying learning and decision processes in both of these tasks. These kinematic parameters (including joystick speed and displacement, Fréchet similarity of trajectories, tortuosity, angular standard deviation, and movement vigor) provide key additional insights into the motor execution of choices that are not as readily assessed in other paradigms. The system's flexibility of task design, joystick readout, and ease of construction represent an advance compared with currently available manipulandum tasks in mice. We provide detailed schematics for constructing the setup and protocols for behavioral training using both paradigms, with the hope that this open-source resource is readily adopted by neuroscientists interested in mechanisms of sensorimotor integration, motor control, and choice behavior.

## Significance Statement

Behavioral paradigms for experiments in head-restrained mice are important for investigating the relationship between neural activity and behavior. However, behavioral setups are often constrained by high cost, design complexity, and implementation challenges. Here, we present an open-source platform for behavioral training of head-fixed mice using a joystick manipulandum. The setup allows for the creation of multiple behavioral paradigms, including an auditory-motor discrimination paradigm, and a value-based decision-making task. We include detailed instructions for construction and implementation of the entire open-source behavioral platform.

## Introduction

A major goal of neuroscience is to understand the relationship between neural activity and behavior. Development of sophisticated behavioral paradigms for experiments in head-restrained mice has received considerable effort because of the ability to measure and manipulate neural activity in a genetically tractable mammalian species. However, the creation of such paradigms is often constrained by high costs, design complexity, and implementation challenges. The rise of open-source approaches in neuroscience has begun to address these barriers (Burgess et al., 2017; Mathis et al., 2017; Bollu et al., 2019; Belsey et al., 2020; Wagner et al., 2020; Manita et al., 2022; Forghani et al., 2023; Gordon-Fennell et al., 2023; Ozgur et al., 2023), making diverse behavioral paradigms more widely available for studying the neural basis of behavior.

Head-fixed behaviors in mice, while limited in their naturalistic scope, offer significant advantages for studying behavior in a controlled and repeatable environment (Guo et al., 2014). These setups allow researchers to precisely combine the delivery of sensory cues with the measurement of motor outputs, providing a robust framework for implementing multiple behavioral paradigms (Bjerre and Palmer, 2020). Such paradigms include Go/No-Go tasks (Guo et al., 2014; Micallef et al., 2017; Helmchen et al., 2018), two-alternative forced-choice (2AFC) tasks (Mayrhofer et al., 2013; Guo et al., 2014; Burgess et al., 2017; Estebanez et al., 2017; Morandell and Huber, 2017; Gilad et al., 2018; Belsey et al., 2020; Ozgur et al., 2023; Pan-Vazquez et al., 2024), working memory assessments (Gilad et al., 2018; Inagaki et al., 2019), and locomotion or exploration tasks (Kislin et al., 2014; Nashaat et al., 2016; Mosberger et al., 2024). The tasks utilize a range of motor outputs, including licks (Guo et al., 2014; Micallef et al., 2017; Gilad et al., 2018; Helmchen et al., 2018; Inagaki et al., 2019; Ozgur et al., 2023), reaching platforms (Estebanez et al., 2017), and floating environments (Kislin et al., 2014; Nashaat et al., 2016). In addition, manipulanda such as turning wheels (Burgess et al., 2017; Pan-Vazquez et al., 2024) and joysticks (Mathis et al., 2017; Morandell and Huber, 2017; Belsey et al., 2020; Yang and Masmanidis, 2020; Mosberger et al., 2024; Nicholas and Yttri, 2024) provide access to fine-grained kinematic information in a head-fixed context, allowing for detailed dissection of neural activity and effects of optogenetic manipulations. This level of control makes head-fixed paradigms with manipulanda invaluable for dissecting the relationship between neural activity and behavior.

Recent advances have demonstrated the utility of joystick manipulanda with high spatiotemporal precision in studying motor behavior (Belsey et al., 2020; Wagner et al., 2020; Inoue et al., 2021), including reaching tasks (Estebanez et al., 2017; Miri et al., 2017; Bollu et al., 2019; Park et al., 2022; Contreras-López et al., 2023; DeWolf et al., 2024; Nicholas and Yttri, 2024), long-term motor learning (Hwang et al., 2019, 2021), reinforcement learning (Panigrahi et al., 2015; Yttri and Dudman, 2016; Roth et al., 2024), motor exploration and refinement (Mosberger et al., 2024), sensory discrimination (Hwang et al., 2017; Yang and Masmanidis, 2020; Franco and Goard, 2024), and vibrotactile sensory-motor integration (Estebanez et al., 2017; Morandell and Huber, 2017). Despite these advantages, joysticks have not been widely adopted, due in part to design complexity, high costs (with notable exceptions, such as Belsey et al., 2020; Ozgur et al., 2023), and a lack of modularity. Addressing these barriers is essential for improving accessibility and promoting the widespread use of joystick-based paradigms in neuroscience.

In this work, we present an open-source joystick platform designed to provide modularity and flexibility for diverse behavioral tasks, which we demonstrate through two novel paradigms. The first is a 2AFC auditory-motor discrimination task in which mice push or pull the joystick to report different tones. The second is a value-based decision-making task that examines decision-making strategies and value-related motor output through joystick manipulation. In contrast with existing joystick-based rigs in the field (Belsey et al., 2020; Wagner et al., 2020; Ozgur et al., 2023; Mosberger et al., 2024), our setup features a fixed-base horizontal joystick with two axes of movement in the forward–backward and upward–downward directions. The setup is also compatible with a bar to restrict joystick motion to the forward–backward dimension, thus facilitating training. In addition, our joystick can be mounted on an affordable servo motor to enable joystick presentation and retraction, limiting the mouse's interaction with the joystick to specified time windows of behavioral trials. Our joystick platform thus adds to the literature a cost-effective and versatile solution for investigating motor control and decision-making.

## Material and Methods

### Behavior rig hardware

**Figure 1.**
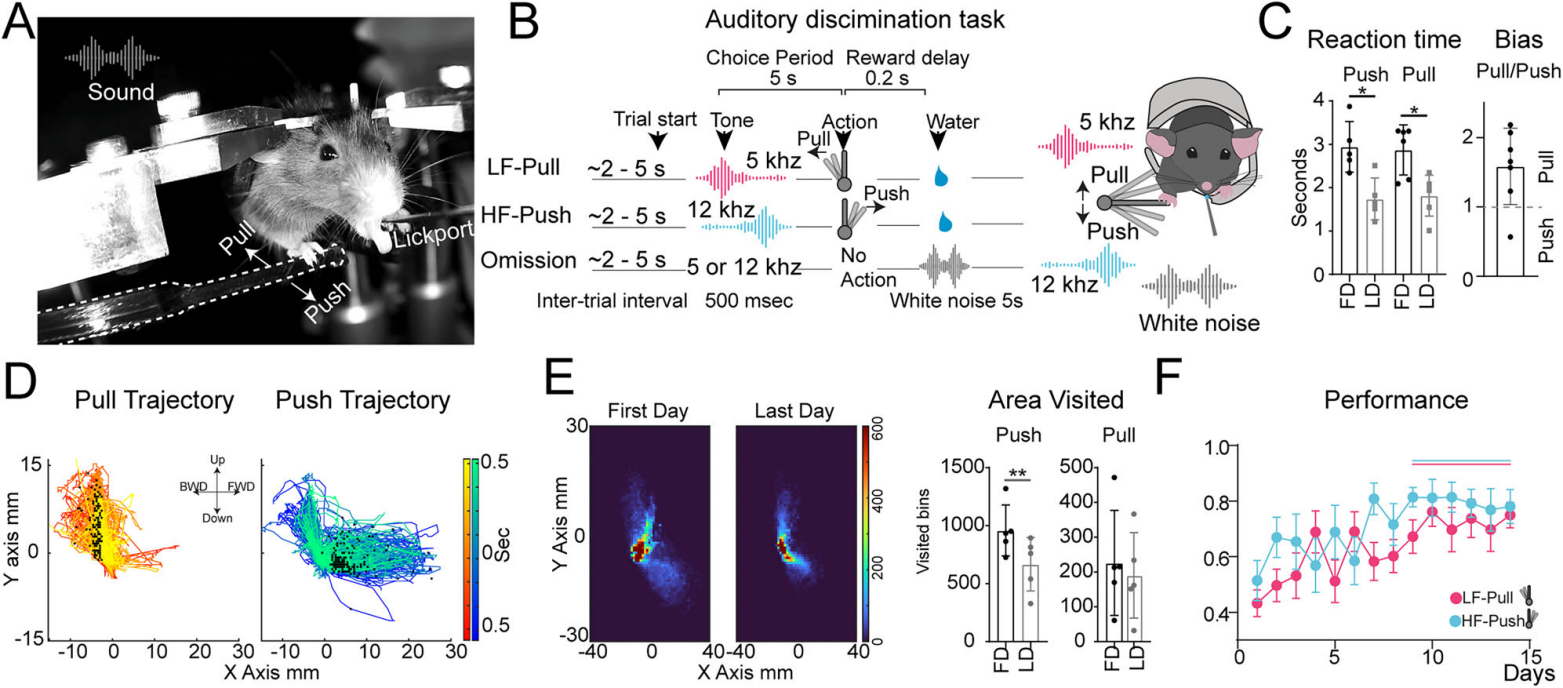
Mice learn to discriminate between distinct sounds and corresponding actions while reducing exploratory push trajectory. ***A***, Representative photo of an animal pushing the joystick after a tone. ***B***, Task schematic: mice undergo a 2–5 s intertrial interval and then hear a 500 ms high- or low-frequency tone. Correct joystick pulls or pushes yield a water reward; omissions trigger a 5 ms white noise. ***C***, Reaction time decreases with training for both displacements. Reaction time is defined as the time from sound onset to joystick onset displacement, including a 1 s grace period. Pull/push ratio shows mice initial motion preference to a specific displacement. ***D***, Joystick displacement around the choice threshold shows pulls as upward–backward and pushes as downward–forward movements. ***E***, Joystick area visited on first day and last day: warmer colors show higher frequency. Push area decreases with training. ***F***, Performance improves over time, measured as the proportion of correct trials. *n *= 10. Vertical solid lines indicate *p *< 0.05. **p < 0.0075.

The hardware setup can be configured for both the auditory-motor discrimination task and the value-based decision-making task, but it consists of the same basic components that can be adjusted as needed. These include Mouse Head Plates and Holder (Janelia HHMI Head Plate and Holder; https://hhmi.flintbox.com/technologies/c04b8f01-f188-472a-b660-368a5f8549ad), a restraining tube (Wagner et al., 2020), a fixed or retractable joystick, a speaker, a water spout, and a licking sensor (Fig. 1*A*,*F*). 3D models for both the fixed and retractable joystick are available in the supplementary materials, along with a step-by-step assembly guide.

**Movie 1.** Example of a head-fixed mouse performing the auditory-motor discrimination task. The video shows separate trials where the animal pushes or pulls a joystick in response to high- or low-frequency tones, respectively. [View online]

**Movie 2.** Example of a head-fixed mouse performing the value-based decision-making task using a retractable joystick. [View online]

The system was designed for ease of assembly, with a focus on reproducibility and scalability, allowing for creation of multiple setups within the same lab. Cooler boxes or cabinets with soundproof foam are used as chambers, while the setup is assembled using Thorlabs components, such as breadboards, optical posts, and clamps (Extended Data Figs. 1, 5). This design enables modularity and flexibility to create multiple rigs—either enclosed or open—as needed for experiments involving optogenetics, calcium imaging, or fiber photometry.

The restraining tube connects to a Thorlabs clamp and an optical post, allowing height adjustments to align precisely with the head plate holders (Extended Data Figs. 1, 7, 8). The 3D-printed joystick system consists of two or three laser-printed parts, depending on whether the joystick is fixed in one position or retractable via a motor. The primary part is an 8 cm stick, which reduces the force required for displacement, and is glued to a 3D-printed base that secures both the stick and a 5VDC two-axis analog APEM thumbstick for measuring motion displacements (Extended Data Figs. 1, 6).

10.1523/ENEURO.0038-25.2025.f1-1 Extended Figure 1 Download Extended Figure 1, TIF file.

10.1523/ENEURO.0038-25.2025.f2-1 Extended Figure 2 Download Extended Figure 2, TIF file.

10.1523/ENEURO.0038-25.2025.f3-1 Extended Figure 3 Download Extended Figure 3, TIF file.

10.1523/ENEURO.0038-25.2025.f4-1 Extended Figure 4 Download Extended Figure 4, TIF file.

10.1523/ENEURO.0038-25.2025.f5-1 Extended Figure 5 Download Extended Figure 5, TIF file.

10.1523/ENEURO.0038-25.2025.f6-1 Extended Figure 6 Download Extended Figure 6, TIF file.

10.1523/ENEURO.0038-25.2025.f7-1 Extended Figure 7 Download Extended Figure 7, TIF file.

10.1523/ENEURO.0038-25.2025.f8-1 Extended Figure 8 Download Extended Figure 8, TIF file.

10.1523/ENEURO.0038-25.2025.f9-1 Extended Figure 9 Download Extended Figure 9, TIF file.

10.1523/ENEURO.0038-25.2025.f10-1 Extended Figure 10 Download Extended Figure 10, TIF file.

In the fixed joystick configuration, an additional 3D-printed part supports the joystick on one end and an adjustable friction magic arm on the other. This magic arm allows effortless positioning of the joystick, ensuring it consistently aligns below the mouse's paw on the right side of the restraining tube. The opposite end of the magic arm attaches to an optical post mounted on a Thorlabs breadboard (Extended Data Fig. 2).

The retractable joystick setup requires two additional 3D-printed parts. The first, a reel holder, supports the joystick and enables it to slide along the second part, the servo holder, which is connected to a servo motor. This setup provides a cost-effective retractable system that can be easily controlled with a microprocessor and records joystick motion displacements (Extended Data Fig. 6).

The water spout consists of a 20 G needle with a cut and smoothed tip, connected to tubing on one side of a solenoid valve (Parker: 003-0218-900 or Lee Company: LHDA1231115H) designed for silent water dispensing. The valve is connected to a tube leading to a 50 ml syringe that serves as the water reservoir. In the auditory-motor discrimination setup, the water spout is held in place by a magnetic arm and clamp attached to a steel base, which also holds a night-vision camera and a speaker (Extended Data Fig. 3). In the value-based decision-making setup, the water spout is integrated with an infrared lickometer (Sanworks: 1020) that is held in place by a steel arm and clamp attached to a steel base (Extended Data Fig. 9).

To control the auditory-motor discrimination task, we use two Arduino Uno microprocessors. The first Arduino connects to the joystick for continuous recording of motion displacements while maintaining communication with the second Arduino, which manages the entire task. Arduino 2 controls the FX sound board to play custom audio, the lick sensor through an MPR121 capacitive sensor, and the water solenoid valve via an H-bridge, allowing control of an external 12 V power source. Additionally, a button is included to start the task as desired (Extended Data Fig. 4).

To control the value-based decision-making task, we use two Arduino microprocessors. An Arduino Mega hosts the main behavioral code, which records real-time joystick position and licks, and manages a box light, a GO cue light, and a solenoid valve (via a 12 V power supply and H-bridge). It communicates with an Arduino Uno to generate pseudo-white noise via a speaker (Extended Data Fig. 10), which is used as a punishment signal (Extended Data Fig. 11).

This setup, while designed for the two presented tasks, can be adjusted for other task configurations by adding different components. For example, a second water port could be added for two-choice decision-making, or the sensory modality could be modified to include olfactory, visual, or whisker stimulation with minimal adjustments. This setup has also been used for calcium imaging, optogenetics, and fiber photometry (data not shown), allowing the addition of multiple transistor-transistor logic (TTL) signals to control a microscope or other devices needed for various behavioral experiments.

### Behavioral task software

#### Auditory-motor discrimination task

For the auditory-motor discrimination task, we use a primary Arduino to record joystick displacement as analog input voltage, detected through changes in resistance across two potentiometers on the *x*- and *y*-axes. The Arduino's 10 bit analog-to-digital converter (ADC) interprets analog voltage values from 0 (0 volts) to 1,023 (5 volts). The joystick centers ∼500 units for both axes, with displacements ranging from 9.8 to 24.5 mV, corresponding to changes of 2–5 units, and an “X” mm displacement is registered as a push or pull depending on the direction. When the threshold is reached, the primary Arduino sends a TTL signal to the secondary Arduino, which is recorded as a response from the mouse. The joystick response is sent via serial communication to be displayed and saved using a custom Python code.

The task is hardcoded on the second Arduino, which manages the sound board, joystick push/pull inputs, intertrial interval, sound card, and the lick sensor (using a capacitive sensor). Task events are displayed and saved as a text file through the Python code. Once the programs are uploaded, both Arduinos await a TTL signal from a physical button wired to the second Arduino to start the task. This TTL signal triggers simultaneous acquisition on both Arduinos and any additional TTLs used for two-photon imaging, optogenetics, or fiber photometry. All code for data collection and analysis can be found on the Margolis Lab GitHub on the following repository: https://github.com/margolislab/Open-Source-Joystick-Platform.

#### Value-based decision-making task

For the value-based decision-making task, our joystick displacement readings are integrated with our Arduino Mega UART behavior code output. In addition to the joystick displacement processing steps outlined above for the auditory-motor discrimination task, we calibrate these 10 bit ADC output (0–1,023) voltage readings to known displacements from baseline in millimeters on a box-by-box basis. These linear calibration functions are hardcoded into the behavior code and used to convert voltage values to millimeters in real time. We leverage the Arduino Mega's memory and baud rate capacity to generate a 20 ms moving average joystick position in millimeters, to minimize contributions of aberrant spurious reads due to electrical interference. Anteroposterior deviations of >3 mm from baseline are registered as choices. When the joystick is retracted between trials, we generate new baseline reads to account for potential baseline drift.

This Arduino Mega also sends behavioral data to a behavior computer in real time via UART and TTL signals to our photometry and optogenetics systems. In addition, it manages our lick sensor, solenoid valve, house light, and servo motor. A separate Arduino Uno, triggered by the Arduino Mega, is used to generate pseudo-white noise as a signal that the animal has entered a time-out period following an omission or premature choice. All code for data collection and analysis can be found on the Margolis Lab GitHub on the following repository: https://github.com/margolislab/Open-Source-Joystick-Platform.

See Extended Data for clear instructions on how to build it.

## Results

### Mice learn to discriminate between distinct sounds and corresponding actions while refining the kinematic parameters of joystick movements

Here, we introduce a two-alternative forced-choice auditory-motor discrimination task in which animals push or pull a joystick to indicate whether they have heard a high- or low-frequency tone. This setup allows for the analysis of exploratory trajectories, velocity, tortuosity, displacement patterns, and angular motion over the course of learning, providing rich insights into motor behavior and the cognitive processes underlying decision-making. Head-fixed water-restricted mice earn ∼10 μl water rewards by displacing a joystick in response to specific auditory cues. Joystick movements are categorized as anterior (push) or posterior (pull), corresponding to distinct high-frequency (12 kHz) or low-frequency (5 kHz) tones, respectively, each accompanied by five overtones. Reward delivery is controlled via a soundless solenoid valve equipped with a capacitive sensor at the lick spout. Auditory stimuli are presented through a front-mounted speaker controlled by a programmable sound card (Fig. 1*A*). The static joystick is positioned beneath the mouse's right paw, while the left paw rests on a body tube. This setup forces right-paw use, enabling neuronal contributions to be studied through recordings or manipulations on the contralateral or ipsilateral side relative to the movement.

The task begins with a variable pretrial interval of 2–5 s, followed by a 500 ms auditory cue. Mice are given a 5 s window to perform the correct joystick displacement. Correct responses trigger a 200 ms delay before reward delivery, while omissions result in a 5 s white noise signal and a reset intertrial interval (Fig. 1*B*, Extended Data Video 1, Movie 1). Mice are trained in daily sessions consisting of two single-association phases: low frequency–pull and high frequency–push. Both associations are trained each day, with the training order alternating daily. Sessions last for a maximum of 30 min or until 100 rewards are obtained, with expert animals completing 200 correct trials and consuming up to 2 ml of water per day.

With task learning, mice significantly reduce their reaction times, as evidenced by a decrease in the time taken to respond to stimuli (paired *t* test, *p* < 0.05; Fig. 1*C*, left). Additionally, during the initial training sessions—when mice only moved the joystick to obtain a reward—an analysis of the maximum number of joystick displacements revealed that most mice exhibited a preference for pulling rather than pushing (Fig. 1*C*, right).

Joystick movements are recorded in two dimensions (*x-* and *y-*axes), enabling the visualization of motor behavior trajectories. Push and pull actions are color-coded (red for pull, blue for push), and a temporal gradient highlights joystick movements 0.5 s before and after reaching the reward threshold. A black dot marks the point at which a choice was registered as either a push or a pull (Fig. 1*D*). Push actions involve downward–forward joystick displacement, while pull actions are characterized by upward–backward movement. This configuration provides a detailed two-dimensional representation of motor trajectories (Fig. 1*D*).

Exploratory behavior during learning was assessed by defining a workspace for all mice, based on the minimum and maximum *x*- and *y*-coordinates of joystick displacement across all mice. The joystick displacement workspace was binarized into smaller divisions, with each bin measuring 1 mm^2^. The trajectory areas explored were then calculated. Mice (*n* = 6) showed a significant reduction in the area visited during push movements between the first and last day of training (paired *t* test, *p* < 0.0075). In contrast, no significant change was observed in the area visited during pull movements (paired *t* test, *p* = 0.15; Fig. 1*E*).

Performance was evaluated as the ratio of correct trials to the total number of correct trials and omissions. Mice exhibited significant performance improvement after session nine compared with the first day of training (two-way ANOVA, Dunnett's multiple comparison against first day, *p* < 0.05; Fig. 1*F*). Mice learn the auditory-motor association in 15 d, excluding 5 d of experimenter habituation during which the animals get used to handling, 2 d of head-fix habituation during which the mice freely drink water rewards while head-fixed, and 3–5 d of joystick association during which any displacement results in a reward, resulting in a month of training.

The differential reduction in the area visited—defined as how much mice explore the joystick movement—between pushing and pulling can be explained by two factors. First, the physical constraints of the pulling motion likely limit its range. Observations indicate that when pulling, mice tend to move the joystick primarily backward and slightly upward, whereas pushing involves more dynamic movements (down and forward), resulting in a larger initial exploration area.

Second, the initial preference for pulling observed in the mice may have reduced the potential for further refinement. Since mice were already more comfortable with pulling, there was less room for improvement in that motion compared with pushing, which had a greater scope for learning and optimization.

**Figure 2.**
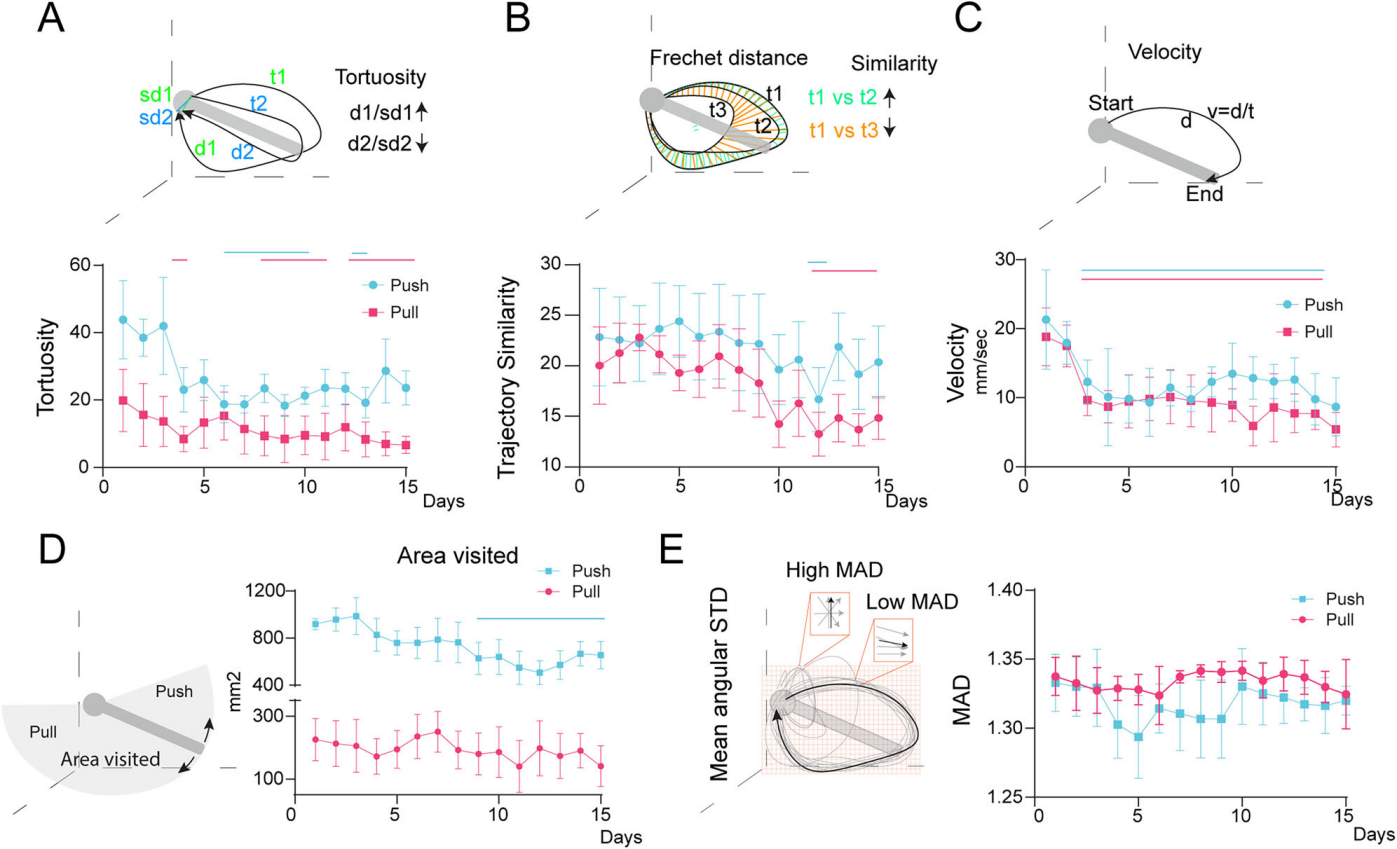
Joystick trajectory dynamics change during auditory-motor integration learning, indicating motor refinement. ***A***, Push and pull tortuosity across training: mice show fewer circuitous routes for both displacements. ***B***, Mean trajectory similarity increases across sessions, indicated by lower Fréchet distance over time. ***C***, Velocity to reach the displacement threshold decreases during the first days of training. ***D***, Joystick exploratory area visits decrease for push displacements as learning progresses. ***E***, Mean angular deviation (MAD) remains stable throughout learning. *n *= 5. Vertical solid lines indicate *p *< 0.05.

To quantify mouse behavior within the joystick workspace, we first identified trial-specific movement trajectories from the session data. Repeated coordinate pairs of the joystick's location were removed, and the coordinates were centered by subtracting the median *x*- and *y*-coordinates. Each trajectory was labeled as either a push or pull trial. These trajectories were then used to calculate the average tortuosity, where higher values indicate a longer, more circuitous route from the starting position to the point of maximum displacement and back. Tortuosity was computed as the ratio of the total path length to the Euclidean distance between the first and last points in the trajectory (Mosberger et al., 2024). Session averages of tortuosity revealed that mice initially exhibited high tortuosity, which decreased and stabilized as they became proficient in the task (two-way ANOVA, Dunnett's multiple comparison against first day, *p* < 0.05; Fig. 2*A*).

To further analyze joystick displacement dynamics, we compared the pairwise similarity of joystick trajectories using the Fréchet distance (Ursell, 2025). For each session, we evaluated joystick displacement and measured the similarity across all possible combinations of trajectories and calculated a mean value per session. Results showed that mice increased movement consistency over time, reflected by a significant reduction in the Fréchet similarity index (two-way ANOVA, Dunnett's multiple comparison against first day, *p* < 0.05; Fig. 2*B*).

We also measured the velocity of joystick motions, defined as the distance between the first point and the point of maximum displacement divided by the corresponding time interval. Mice demonstrated a significant increase in movement velocity compared with their performance on the first day of training (two-way ANOVA, Dunnett's multiple comparison against first day, *p* < 0.05; Fig. 2*C*).

To define the joystick workspace, we calculated the minimum and maximum *x*- and *y*-coordinates across all animals. The explored area within this workspace was quantified by binning the joystick coordinates using MATLAB's “histcounts2” function, as described by Mosberger et al. (2024). Each bin measured 1 mm by 1 mm. The total explored area was calculated by summing the number of visited bins. Over successive training sessions, mice showed a significant reduction in the area explored, which eventually stabilized at a lower value, indicating reduced exploratory behavior only for the push displacement (two-way ANOVA, Dunnett's multiple comparison against first day, *p* < 0.05; Fig. 2*D*).

To assess directional consistency, we calculated the mean angular deviation for both push and pull motions using the CircStat MATLAB Toolbox for circular statistics (Berens, 2009). Mean angular deviation was calculated by taking the average of the angular deviation for the angles in each bin for each session. Angular deviation, ranging from 0 to √2, represents variability in directional movements, with higher values indicating greater variability. The angular deviation remained stable throughout training (Fig. 2*E*).

Together, these results demonstrate that mice effectively moved the joystick in two distinct directions, decreased trajectory tortuosity, increased movement similarity and velocity, and refined their displacement strategy by reducing the explored workspace. This evidence supports the task as a robust tool for analyzing motor output dynamics, offering high-quality, detailed behavioral data.

This auditory-motor discrimination task provides a robust framework for studying neural and behavioral mechanisms underlying auditory-motor associations, offering key insights into sound-driven action selection and motor learning.

### Joystick kinematic parameters reflect total and relative value in value-based decision-making task

Here, we describe a two-armed bandit, joystick-based value-based decision-making task in mice that allows for the study of value-based modulation of motor execution, in addition to recapitulating known characteristics of conventional value-based 2-AFCs.

**Figure 3.**
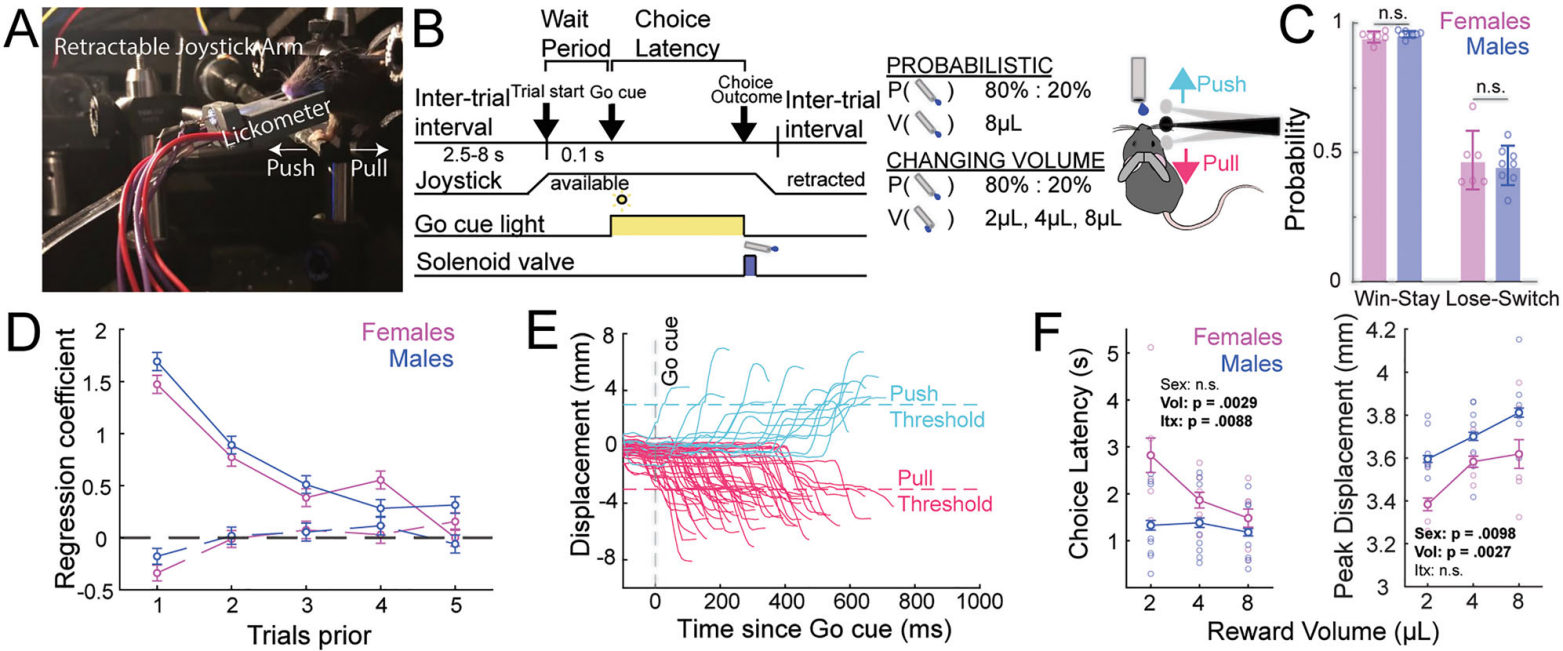
Mice integrate reward evidence across trials to guide next-trial choice and motor vigor. ***A***, Photograph of our behavioral setup. ***B***, Behavioral schematic highlighting two task variants. ***C***, Males (*n* = 8) and females (*n* = 6) prior outcome to guide next trial strategy. ***D***, Logistic regression weights of prior trial rewarded (undashed) and unrewarded (dashed) outcomes for predicting current trial repetition of prior choice. ***E***, Raw joystick choice data. ***F***, Demonstration that varying reward volume significantly shapes motor vigor, as measured by peak joystick displacement and, predominantly in females, choice latency.

Head-fixed water-restricted mice obtain 10% sucrose solution rewards via anterior or posterior displacement of a retractable joystick. Reward is delivered via an optical lickometer setup that also tracks licking (Fig. 3*A*). At trial start, the joystick is made available to the mouse via anterior motion of the servo motor. Following a subsequent 100 ms wait period, a GO cue light on the lickometer signals the start of a 10 s window during which the mouse can register a choice via anterior (push) or posterior (pull) displacement of the joystick (Fig. 3*B*, Extended Data Video 2, Movie 2). There are four possible outcomes of a trial: (1) rewarded trial, followed by 2.5–8 s intertrial interval; (2) unrewarded trial, signaled by turning off house light, followed by 2.5–8 s intertrial interval; (3) omission, signaled by white noise, turning off house light, and 15 s time-out; and (4) premature trial in which mouse registers choice before Go cue, signaled by white noise, turning off house light, and 15 s time-out.

Mice are trained in a sequential behavioral paradigm consisting of (1) probabilistic reversal and (2) changing volume phases (Fig. 3*B*). The probabilistic reversal phase consists of blocks in which one of two choices is more likely to be rewarded than the other (push blocks and pull blocks), with reward probabilities of 80%:20%. Each block has a minimum duration of 17 rewarded trials plus a geometrically distributed random variable (*p *= 0.4), after which the high reward probability side is reversed in an un-cued manner. As in the auditory-motor discrimination task, mice only register choices with their right forepaw. Mice in this phase integrate prior-trial evidence to guide next-trial decisions as evidenced by win-stay/lose-switch analysis as well as logistic regression of choice and reward history (Fig. 3*C*,*D*). The changing volume phase builds on this task structure by adding reward volume as an additional parameter that varies by block, using reward volumes of 2, 4, and 8 μl. Mice register choices with varying latencies and peak joystick displacement (Fig. 3*E*). We find that, in higher total value contexts, mice register choices with shorter choice latency and higher peak joystick displacement (Fig. 3*F*). Unlike choice latency, the peak displacement phenotype is robust across males and females, suggesting that a joystick-based design offers unique, key insights into animals’ regulation of motor vigor based on total value as compared with conventional, binarized lever press- or lick-based tasks (Wang et al., 2013; Alabi et al., 2020).

An advantage of our joystick-based value-based decision-making paradigm over conventional lever-press or lick-based paradigms in mice is the ability to read out kinematic parameters of operant choice. Vigor is known to reflect real-time internal value representations (Takikawa et al., 2002; Shadmehr et al., 2019).

**Figure 4.**
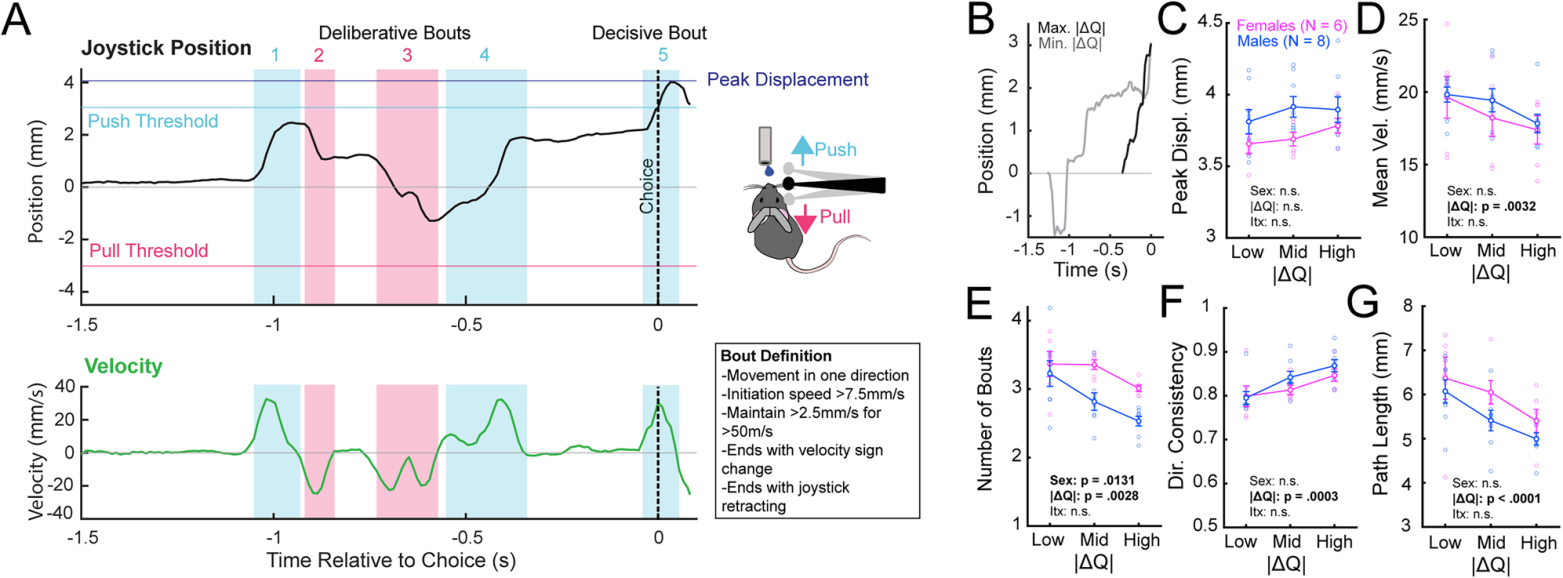
Readouts of joystick trajectory in different relative value contexts reflect animal uncertainty. ***A***, Segmentation of joystick position trace into movement “bouts” based on joystick velocity. ***B***, Representative traces from high uncertainty (low |Δ*Q*|) and low uncertainty (high |Δ*Q*|) contexts. In higher-uncertainty contexts, mice register choices with greater mean velocity (***D***), greater number of movement bouts (***E***), lower consistency of direction of joystick motion across bouts (***F***), and greater path length (***G***). Peak joystick displacement is not significantly affected by degree of uncertainty (***C***). *p < 0.05; **p < 0.01; ***p < 0.001.

In this task, we find that mice's joystick trajectories often exhibit extensive deliberation before crossing choice threshold (Fig. 4*A*). Anteroposterior joystick position relative to baseline can be plotted as a function of time and segmented into movement bouts to capture these deliberative movement bouts leading up to a threshold-crossing decisive bout. We defined bouts according to the following criteria: (1) movement is in one direction, (2) initiation speed is >7.5 mm/s, (3) speed is maintained at >2.5 mm/s for >50 ms, (4) bout ends with velocity sign change or joystick retracting. Of note, unlike the auditory-motor discrimination task, our value-based decision-making task constrains motion to the anteroposterior axis via addition of a metal bar underneath the joystick, limiting up-down joystick displacement.

A range of kinematic parameters can be extracted from joystick movement traces. Peak displacement is defined as the maximum extent of displacement of the joystick away from baseline on a given trial. Number of bouts is defined as the number of movement bouts the animal initiates in a given trial. Directional consistency is the proportion of these bouts that occur in the higher frequency direction (Eq. 1), with a value of 1 implying that all bouts occur in one direction. Mean velocity is defined as the mean velocity of the joystick on the decisive bout. Path length is defined as the distance travelled by the joystick in any direction on a given trial.

Directionalconsistency=n(boutsinmostfrequentdirection)/n(boutsinalldirections).

We found that our readouts of joystick trajectory reflect animals’ internal representations of certainty that one joystick direction is more likely to yield reward than another, i.e., relative value. Using a two-parameter Q-learning model with nondifferential forgetting (Ito and Doya, 2015; Choi et al., 2023), we generated trial-by-trial estimates of animals internal representation of the value of push and pull actions (*Q*_push_ and *Q*_pull_). We computed the absolute value of the difference between *Q*_push_ and *Q*_pull_ (Δ*Q* = *Q*_push_ − *Q*_pull_) to gauge the animals’ experienced uncertainty on a given trial, where low |Δ*Q*| implies more similar value representations of push and pull actions, and therefore greater uncertainty regarding which choice is more likely to be rewarded. Movement trajectories in high |Δ*Q*| (low uncertainty) and low |Δ*Q*| (high uncertainty) contexts are distinct, as is illustrated in example traces (Fig. 4*B*). In lower |Δ*Q*| trials, we found that animals trended toward lower peak displacement (Fig. 4*C*) and had significantly greater mean joystick velocity (Fig. 4*D*), a significantly greater number of joystick movement bouts (Fig. 4*E*), significantly lower directional consistency (Fig. 4*F*), and significantly greater path length (Fig. 4*G*), reflective of greater uncertainty. Our joystick kinematic parameters thus provided a key additional insight into animals’ dynamic representations of relative value.

## Discussion

Our work documents an open-source platform for behavioral training of head-fixed mice using a forelimb-based joystick manipulandum. We demonstrate, in a novel 2AFC auditory-motor discrimination paradigm, that mice refine their motor execution throughout learning. Similarly, as mice learned a novel value-based decision-making task, they constrained their motor vigor in the context of lower reward volume and uncertainty. In these tasks, the joystick provided kinematic readouts reflective of learning stage and internal value representations. Joystick kinematic parameters thus provided key additional insights into the motor execution of choices that are not as readily assessed in freely moving, lever press, or head-fixed licking contexts.

Disentangling sensory neuronal encoding from motor output is complex yet crucial for decision-making (Mohan et al., 2018; Ranieri et al., 2022). While some brain areas are primarily involved in sensory processing, others, including the striatum, serve as integrative hubs for both sensory and motor inputs (Gerfen, 1984; Hunnicutt et al., 2016), with individual neurons often receiving both sensory- and motor-related synaptic input (Ramanathan et al., 2002; Assous and Tepper, 2019; Lee et al., 2019; Sanabria et al., 2024). In this context, paradigms that disentangle sensory inputs from motor outputs are essential for identifying their distinct contributions to neuronal activity (Burgess et al., 2017; Estebanez et al., 2017; Morandell and Huber, 2017; Ozgur et al., 2023). While simpler sensory discrimination paradigms, such as Go/NoGo licking tasks, can confirm an animal's ability to distinguish stimuli, they provide limited insight into the decision-making process beyond sensory discrimination (Guo et al., 2014). 2AFC behavioral paradigms can be used to explore perceptual decision-making (Bjerre and Palmer, 2020). By presenting two stimuli and associating them with two distinct actions, these paradigms allow for the generation of different behavioral metrics to compare and contrast against neuronal activity. This approach facilitates testing for selectivity and distinguishing between sensory stimulus, choice, motor action, and outcome selection (Chen et al., 2024). Here, we introduce a 2AFC auditory-motor discrimination paradigm that incorporates custom sounds—tones with overtones at high and low frequencies. Mice are trained to discriminate between these sounds and report their choices through distinct joystick movements: anterior (push) or posterior (pull) displacements executed with a single forepaw. Our paradigm could be modified easily to include other sensory cues (e.g., visual, tactile, olfactory) relevant for investigating sensory discrimination and cued movements in multiple modalities.

A joystick-based task design also offers significant advantages in the study of value-based decision-making. In addition to recapitulating aspects of known features of two-armed bandit designs, including integration of evidence across trials and adaptation of behavior as contingencies change (Tai et al., 2012; Parker et al., 2016; Chantranupong et al., 2023), it enables the study of value-related invigoration of movements as is seen classically with saccades in primate value-based decision-making designs (Takikawa et al., 2002; Reppert et al., 2015; Shadmehr et al., 2019). In addition, trial-level joystick traces reveal change-of-mind decisions (Stone et al., 2022) on several high-uncertainty trials (Fig. 4*A*,*B*), making our task a valuable platform for investigating the neural basis of these decisions that are observed in primates in the setting of uncertainty (Resulaj et al., 2009; Atiya et al., 2020) or low confidence (Sanders et al., 2016). We demonstrate that our joystick kinematic metrics, such as peak displacement, mean velocity, path length, and properties of movement bouts, are reflective of animals’ internal total and relative value representations, as captured by standard reinforcement learning algorithms. Given the intricate interplay of value- and vigor-related information in the cortex, basal ganglia, and the midbrain (Nakamura and Hikosaka, 2006; Niv et al., 2007; Wang et al., 2013; Hikosaka et al., 2014; Dudman and Krakauer, 2016; Shadmehr et al., 2019), this task provides rich behavioral outputs with which to study the neural representation of value-based decision-making in mice.

We present two distinct behavioral paradigms built on a shared hardware design, offering a versatile framework adaptable to diverse experimental needs. These paradigms can be modified to accommodate different sensory modalities by altering the stimuli. For example, whisker stimulation can be implemented using a 12 V stepper motor paired with an Adafruit motor shield for Arduino and 3D-printed windmill textures. Similarly, visual stimulation can be introduced using an Adafruit SSD1327 OLED Graphic Display interfaced with Arduino via I2C. Additionally, a simple sensory discrimination paradigm can be incorporated through optogenetic stimulation of sensory inputs triggered by Arduino transistor-transistor logic signals (TTLs; Sachidhanandam et al., 2013). The H-bridge used to drive the water solenoid is designed to support an additional solenoid. This feature enables the integration of a second water spout, facilitating the development of a head-fixed version of a two-step task (Akam et al., 2021) or devaluation paradigms (Turner and Balleine, 2024). Because our setup operates using Arduinos, it can easily interface with fiber photometry, optogenetics, or 1/2-photon calcium imaging via TTLs, facilitating study of the neural basis of behavior.

A limitation with head-fixed, joystick-based setups is their relative difficulty. Head fixation per se can delay learning timelines as it can increase animal anxiety and is less “naturalistic.” In addition, while mice readily learn to displace the joystick manipulandum within a couple of days, it is anecdotally more difficult for mice to learn to distinguish two different directions. This part of training requires attention and can take up to a month, as is also seen in other joystick-based paradigms (Hwang et al., 2021). One way to expedite training is to constrain joystick motion using bars to minimize out-of-plane motion or force the mouse to move the joystick in a nonpreferred direction (i.e., forcing a mouse to pull that prefers to push). Another potential limitation is that our joystick comes in from the right side and cannot be displaced along the left-right axis. It is therefore not ideal in the study of left versus right choice as is seen in some basal ganglia studies (Tai et al., 2012; Bolkan et al., 2022), which would require left-right joystick designs (Belsey et al., 2020). Another limitation is that the stiffness of our joystick cannot be adjusted in real time during behavior, which restricts its use in studying cost or effort-based decision-making. Alternative approaches to this problem include (1) dynamically adjusting the joystick displacement threshold for reward or (2) using joysticks of different lengths to modify resistance across sessions—longer joysticks are easier to move than shorter ones. Additionally, joysticks can be fitted with different springs to alter resistance, with looser springs requiring less effort than tighter ones (as described by Bollu et al., 2019 and Belsey et al., 2020). Where the kinematics of action execution are not of interest, head-fixed licking-based paradigms or freely moving lever/nose-poke based paradigms should be preferred as these might be more readily learnable.

Investigation of the neural processes underlying motor control requires precise behavioral readouts that capture the kinematics of motor actions. Here, we present a low-cost, open-source, joystick-based platform for the behavioral training of head-fixed mice, which allows for the study of learning and task-related refinement in motor execution. The joystick metrics we highlight provide only a glimpse into the wealth of spatiotemporal data that can be extracted from our real-time joystick position recordings. We hope this setup will be readily adopted and expanded upon by the neuroscience community to provide insights into the kinematic parameters of sensorimotor integration, decision-making, value representation, and other neural processes.

## Synthesis

Reviewing Editor: Mark Laubach, American University Decisions are customarily a result of the Reviewing Editor and the peer reviewers coming together and discussing their recommendations until a consensus is reached. When revisions are invited, a fact-based synthesis statement explaining their decision and outlining what is needed to prepare a revision will be listed below. The following reviewer(s) agreed to reveal their identity: Mostafa Nashaat.

Thank you for sending your paper to eNeuro. It was reviewed by two experts. They viewed the paper most favorably but did ask for some revisions to be made. Their reviews are given below in full.

Reviewer 1

Advances the Field:

This is an excellent paper describing the build and characterization of a head-fixed apparatus for learning auditory and value associations. It will make it easier and more affordable for many labs to perform these types of experiments.

Comments to the Authors:

The authors present a compelling description of an open source joystick/lick-port, capable of testing auditory associations and value-based associations. The device has been well-characterized, and build instructions are very clear and complete for the most part. The manuscript is thorough, and very well-written. In particular, the authors are to be commended on their detailed circuit diagrams showing Arduino connectivity. The manuscript would benefit from some minor changes and additions, outlined below:

1. Why do the authors think mice were more spatially efficient on the last day of push trials but not pull trials, as shown in Figure 1D?

2. In the supplemental sections, the following assembly instructions state "To assemble the Head-Fix setup, you will use a 3D-printed body (as described by Wagner et al.,2020), a tube, and the Janelia HHMI head post assembly". Please include the detailed information from these references, including all print design files, parts list, and step by step assembly instructions.

3. For each of the set-ups, please include a spreadsheet or table itemizing the components needed, including Company and part/catalogue number. The authors have included one such table, but certain items required for the build are missing (for instance, epoxy).

4. Please include example data generated for each of the set-ups, along with the analysis code used to generate the figures.

Reviewer 2

Advances the Field:

This manuscript presents an open-source joystick-based behavioral platform that leverages forelimb motor control in head-fixed mice to study sensory-based (auditory) decision-making and value-based decision-making. The system includes a fixed or retractable joystick, a sound presentation system, a capacitive lick sensor, and a water reward dispenser, allowing for diverse behavioral paradigms. The authors highlight two key tasks: (1) a two-alternative forced-choice (2AFC) auditory-motor discrimination task, where mice push or pull the joystick in response to high- or low-frequency tones, and (2) a value-based decision-making task, where mice adapt their joystick movements based on prior reward history. By analyzing joystick kinematics, the system captures fine-grained motor parameters such as trajectory similarity, velocity, tortuosity, and vigor, offering insights into learning, decision-making, reward expectation, and uncertainty-related movement patterns. Compared to existing paradigms, this low-cost, customizable, and open-source system provides a versatile tool for neuroscience research, with potential applications in optogenetics, calcium imaging, and reinforcement learning studies.

The authors introduce a clear and accessible approach for replicating a joystick-based behavioral platform. The authors demonstrate that the system is versatile and can be applied to various sensory and value-based decision-making paradigms. Unlike traditional lick right/left two-alternative forced choice (2AFC) paradigms, this system provides a detailed analogue readout of joystick trajectories, which may be used to infer the internal state of mice during behavior. Overall, this paper makes a significant contribution by offering a valuable, open-source, low-cost, and versatile behavioral platform for studying decision-making in head-fixed animals, and it can be effectively combined with classical cellular recording and optical perturbation methods.

Comments to the Authors:

1. Software accessibility

To enhance the usability and reproducibility of the method, I suggest organizing and sharing key components of the system more transparently. Specifically, providing design files for 3D-printed parts and the Python software used to implement the different task designs via GitHub would make the platform more user-friendly and easier to replicate. This open-access approach would greatly benefit researchers looking to adopt or modify the system for their own studies.

EDITOR'S COMMENT: You included open-source as a keyword, so please do address this issue.

2. Multimedia

Providing a supplementary video will be very useful to understand the details of the system development and task structure.

EDITOR'S COMMENT: Strong agreement! Please submit a video as extended data.

3. Reaction time analysis

The study offers a comprehensive characterization of joystick kinematics during sensory-based decision making, including visited bins, velocity, trajectory similarity, and vigor (Figures 1 and 2). However, reaction time is not explicitly analyzed. Given its importance for assessing decision urgency, motor preparation, and response impulsivity, examining reaction time variability would be highly valuable. Analyzing this metric across different learning phases, during steady performance (as shown in Figure 1E), and for both pull and push movements (as presented in Figure 2) could reveal whether reaction time decreases with learning and how it correlates with the dynamics of these actions.

4. Motor costs and pre-existing biases in push vs. pull movements

The study demonstrates that pushing and pulling have distinct biomechanical demands (Figure 1C, D &Figure 2). Additionally, during learning (Figure 1E), mice appear to show a preference for pushing over pulling. It would be valuable to quantify the degree of this bias, as it may become more pronounced when less salient auditory stimuli are introduced. If this preference exists, it could significantly impact learning rates and task performance, particularly in conditions where mice rely on weaker sensory cues. A key question to address is if all mice have bias for push or it differs between mice. This would be simple to describe in their result by plotting a win-stay/lose-switch graph or something similar for push and pull across mice.

5. Connecting joystick movements to internal states and change of mind

The use of joystick movements as a readout of the animal's internal state is a compelling aspect of the study. The authors should consider drawing stronger connections to similar research in primates, particularly in studies related to the field of uncertainty and change of mind (Resulaj et al., 2009; Sanders et al., 2012; Atiya et al., 2020; Stone et al., 2022). These studies explore how motor adjustments reflect evolving decision confidence, and integrating these perspectives would strengthen the interpretation of joystick-based behavior in mice. Technical considerations: manipulating torque and motor costs.

6. Another part to address in the discussion is the potential aspect of manipulating the torque in push and pull mechanics. Adjusting these mechanical properties could allow for:

- Controlling for impulsivity bias by altering the effort required for different movements.

- Studying uncertainty-related movement adjustments, providing insights into how movement vigor relates to confidence or reward expectation.

- Investigating effort-based decision-making, where animals weigh the cost of motor actions against expected rewards.

Incorporating such mechanical modifications would expand the utility of the platform and allow for a more nuanced exploration of motor-related decision-making processes. This technical consideration is only a suggestion if the authors think that their system can address above-mentioned applications.

## References

[B1] Akam T, Rodrigues-Vaz I, Marcelo I, Zhang X, Pereira M, Oliveira RF, Dayan P, Costa RM (2021) The anterior cingulate cortex predicts future states to mediate model-based action selection. Neuron 109:149–163.e7. 10.1016/j.neuron.2020.10.013 33152266 PMC7837117

[B2] Alabi OO, Davatolhagh MF, Robinson M, Fortunato MP, Vargas Cifuentes L, Kable JW, Fuccillo MV (2020) Disruption of Nrxn1α within excitatory forebrain circuits drives value-based dysfunction. Elife 9:e54838. 10.7554/eLife.54838 33274715 PMC7759380

[B3] Assous M, Tepper JM (2019) Excitatory extrinsic afferents to striatal interneurons and interactions with striatal microcircuitry. Eur J Neurosci 49:593–603. 10.1111/ejn.13881 29480942 PMC6507406

[B4] Atiya NAA, Zgonnikov A, O’Hora D, Schoemann M, Scherbaum S, Wong-Lin K (2020) Changes-of-mind in the absence of new post-decision evidence. PLoS Comput Biol 16:e1007149. 10.1371/journal.pcbi.1007149 32012147 PMC7018100

[B5] Belsey PP, Nicholas MA, Yttri EA (2020) Open-source joystick manipulandum for decision-making, reaching, and motor control studies in mice. eNeuro 7:ENEURO.0523-19.2020. 10.1523/ENEURO.0523-19.2020 32094292 PMC7131984

[B6] Berens P (2009) Circstat: a MATLAB toolbox for circular statistics. J Stat Softw 31:1–21. 10.18637/jss.v031.i10

[B8] Bjerre AS, Palmer LM (2020) Probing cortical activity during head-fixed behavior. Front Mol Neurosci 13:30. 10.3389/fnmol.2020.00030 32180705 PMC7059801

[B9] Bolkan SS, et al. (2022) Opponent control of behavior by dorsomedial striatal pathways depends on task demands and internal state. Nat Neurosci 25:345–357. 10.1038/s41593-022-01021-9 35260863 PMC8915388

[B10] Bollu T, Whitehead SC, Prasad N, Walker J, Shyamkumar N, Subramaniam R, Kardon B, Cohen I, Goldberg JH (2019) Automated home cage training of mice in a hold-still center-outreach task. J Neurophysiol 121:500–512. 10.1152/jn.00667.2018 30540551 PMC6397391

[B11] Burgess CP, et al. (2017) High-yield methods for accurate two-alternative visual psychophysics in head-fixed mice. Cell Rep 20:2513–2524. 10.1016/j.celrep.2017.08.047 28877482 PMC5603732

[B12] Chantranupong L, Beron CC, Zimmer JA, Wen MJ, Wang W, Sabatini BL (2023) Dopamine and glutamate regulate striatal acetylcholine in decision-making. Nature 621:577–585. 10.1038/s41586-023-06492-9 37557915 PMC10511323

[B13] Chen S, Liu Y, Wang ZA, Colonell J, Liu LD, Hou H, Tien N-W, Wang T, Harris T, Druckmann S (2024) Brain-wide neural activity underlying memory-guided movement. Cell 187:676–691.e16. 10.1016/j.cell.2023.12.035 38306983 PMC11492138

[B14] Choi K, Piasini E, Díaz-Hernández E, Cifuentes LV, Henderson NT, Holly EN, Subramaniyan M, Gerfen CR, Fuccillo MV (2023) Distributed processing for value-based choice by prelimbic circuits targeting anterior-posterior dorsal striatal subregions in male mice. Nat Commun 14:1920. 10.1038/s41467-023-36795-4 37024449 PMC10079960

[B15] Contreras-López R, et al. (2023) The deep cerebellar nuclei to striatum disynaptic connection contributes to skilled forelimb movement. Cell Rep 42:112000. 10.1016/j.celrep.2023.112000 36656714

[B16] DeWolf T, et al. (2024) Neuro-musculoskeletal modeling reveals muscle-level neural dynamics of adaptive learning in sensorimotor cortex. bioRxiv: 2024.2009.2011.612513.

[B17] Dudman JT, Krakauer JW (2016) The basal ganglia: from motor commands to the control of vigor. Curr Opin Neurobiol 37:158–166. 10.1016/j.conb.2016.02.005 27012960

[B18] Estebanez L, Hoffmann D, Voigt BC, Poulet JFA (2017) Parvalbumin-expressing GABAergic neurons in primary motor cortex signal reaching. Cell Rep 20:308–318. 10.1016/j.celrep.2017.06.044 28700934 PMC5522533

[B19] Forghani R, Goodnight B, Latchoumane CV, Karumbaiah L (2023) AutoRG: an automatized reach-to-grasp platform technology for assessing forelimb motor function, neural circuit activation, and cognition in rodents. J Neurosci Methods 387:109798. 10.1016/j.jneumeth.2023.109798 36682731 PMC10071513

[B20] Franco LM, Goard MJ (2024) Differential stability of task variable representations in retrosplenial cortex. Nat Commun 15:6872. 10.1038/s41467-024-51227-7 39127731 PMC11316801

[B21] Gerfen CR (1984) The neostriatal mosaic: compartmentalization of corticostriatal input and striatonigral output systems. Nature 311:461. 10.1038/311461a0 6207434

[B22] Gilad A, Gallero-Salas Y, Groos D, Helmchen F (2018) Behavioral strategy determines frontal or posterior location of short-term memory in neocortex. Neuron 99:814–828.e7. 10.1016/j.neuron.2018.07.029 30100254

[B24] Gordon-Fennell A, Barbakh JM, Utley MT, Singh S, Bazzino P, Gowrishankar R, Bruchas MR, Roitman MF, Stuber GD (2023) An open-source platform for head-fixed operant and consummatory behavior. Elife 12:e86183. 10.7554/eLife.86183 37555578 PMC10499376

[B25] Guo ZV, et al. (2014) Procedures for behavioral experiments in head-fixed mice. PLoS One 9:e88678. 10.1371/journal.pone.0088678 24520413 PMC3919818

[B26] Helmchen F, Gilad A, Chen JL (2018) Neocortical dynamics during whisker-based sensory discrimination in head-restrained mice. Neuroscience 368:57–69. 10.1016/j.neuroscience.2017.09.003 28919043 PMC5726900

[B27] Hikosaka O, Kim HF, Yasuda M, Yamamoto S (2014) Basal ganglia circuits for reward value-guided behavior. Annu Rev Neurosci 37:289–306. 10.1146/annurev-neuro-071013-013924 25032497 PMC4148825

[B28] Hunnicutt BJ, Jongbloets BC, Birdsong WT, Gertz KJ, Zhong H, Mao T (2016) A comprehensive excitatory input map of the striatum reveals novel functional organization. Elife 5:e19103. 10.7554/eLife.19103 27892854 PMC5207773

[B29] Hwang EJ, Dahlen JE, Hu YY, Aguilar K, Yu B, Mukundan M, Mitani A, Komiyama T (2019) Disengagement of motor cortex from movement control during long-term learning. Sci Adv 5:eaay0001. 10.1126/sciadv.aay0001 31693007 PMC6821459

[B30] Hwang E, Dahlen JE, Mukundan M, Komiyama T (2017) History-based action selection bias in posterior parietal cortex. Nat Commun 8:1242. 10.1038/s41467-017-01356-z 29089500 PMC5663966

[B31] Hwang EJ, Dahlen JE, Mukundan M, Komiyama T (2021) Disengagement of motor cortex during long-term learning tracks the performance level of learned movements. J Neurosci 41:7029–7047. 10.1523/JNEUROSCI.3049-20.2021 34244359 PMC8372014

[B32] Inagaki HK, Fontolan L, Romani S, Svoboda K (2019) Discrete attractor dynamics underlies persistent activity in the frontal cortex. Nature 566:212–217. 10.1038/s41586-019-0919-7 30728503

[B33] Inoue T, Terada S, Matsuzaki M, Izawa J (2021) A small-scale robotic manipulandum for motor control study with rodents. Adv Robot 35:898–906. 10.1080/01691864.2021.1912637

[B34] Ito M, Doya K (2015) Distinct neural representation in the dorsolateral, dorsomedial, and ventral parts of the striatum during fixed- and free-choice tasks. J Neurosci 35:3499–3514. 10.1523/JNEUROSCI.1962-14.2015 25716849 PMC4339358

[B35] Kislin M, et al. (2014) Flat-floored air-lifted platform: a new method for combining behavior with microscopy or electrophysiology on awake freely moving rodents. J Vis Exp 88:e51869. 10.3791/51869 24998224 PMC4209781

[B36] Lee CR, Yonk AJ, Wiskerke J, Paradiso KG, Tepper JM, Margolis DJ (2019) Opposing influence of sensory and motor cortical input on striatal circuitry and choice behavior. Curr Biol 29:1313–1323.e5. 10.1016/j.cub.2019.03.028 30982651 PMC6482065

[B37] Manita S, Ikezoe K, Kitamura K (2022) A novel device of reaching, grasping, and retrieving task for head-fixed mice. Front Neural Circuits 16:842748. 10.3389/fncir.2022.842748 35633733 PMC9133411

[B38] Mathis MW, Mathis A, Uchida N (2017) Somatosensory cortex plays an essential role in forelimb motor adaptation in mice. Neuron 93:1493–1503.e6. 10.1016/j.neuron.2017.02.049 28334611 PMC5491974

[B39] Mayrhofer JM, Skreb V, von der Behrens W, Musall S, Weber B, Haiss F (2013) Novel two-alternative forced choice paradigm for bilateral vibrotactile whisker frequency discrimination in head-fixed mice and rats. J Neurophysiol 109:273–284. 10.1152/jn.00488.2012 23054598

[B40] Micallef AH, Takahashi N, Larkum ME, Palmer LM (2017) A reward-based behavioral platform to measure neural activity during head-fixed behavior. Front Cell Neurosci 11:156. 10.3389/fncel.2017.00156 28620282 PMC5449766

[B41] Miri A, Warriner CL, Seely JS, Elsayed GF, Cunningham JP, Churchland MM, Jessell TM (2017) Behaviorally selective engagement of short-latency effector pathways by motor cortex. Neuron 95:683–696.e11. 10.1016/j.neuron.2017.06.042 28735748 PMC5593145

[B42] Mohan H, de Haan R, Mansvelder HD, de Kock CPJ (2018) The posterior parietal cortex as integrative hub for whisker sensorimotor information. Neuroscience 368:240–245. 10.1016/j.neuroscience.2017.06.020 28642168

[B43] Morandell K, Huber D (2017) The role of forelimb motor cortex areas in goal directed action in mice. Sci Rep 7:15759. 10.1038/s41598-017-15835-2 29150620 PMC5693936

[B44] Mosberger AC, et al. (2024) Exploration biases forelimb reaching strategies. Cell Rep 43:113958. 10.1016/j.celrep.2024.113958 38520691 PMC11097405

[B45] Nakamura K, Hikosaka O (2006) Role of dopamine in the primate caudate nucleus in reward modulation of saccades. J Neurosci 26:5360–5369. 10.1523/JNEUROSCI.4853-05.2006 16707788 PMC6675290

[B46] Nashaat MA, Oraby H, Sachdev RN, Winter Y, Larkum ME (2016) Air-track: a real-world floating environment for active sensing in head-fixed mice. J Neurophysiol 116:1542–1553. 10.1152/jn.00088.2016 27486102 PMC5144720

[B47] Nicholas MA, Yttri EA (2024) Motor cortex is responsible for motor dynamics in striatum and the execution of both skilled and unskilled actions. Neuron 112:3486–3501.e5. 10.1016/j.neuron.2024.07.022 39168128

[B48] Niv Y, et al. (2007) Tonic dopamine: opportunity costs and the control of response vigor. Psychopharmacology 191:507–520. 10.1007/s00213-006-0502-4 17031711

[B49] Ozgur A, Park SB, Flores AY, Oijala M, Lur G (2023) A comprehensive, affordable, open-source hardware-software solution for flexible implementation of complex behaviors in head-fixed mice. eNeuro 10:ENEURO.0018-23.2023. 10.1523/ENEURO.0018-23.2023 37286343 PMC10306125

[B50] Pan-Vazquez A, et al. (2024) Pre-existing visual responses in a projection-defined dopamine population explain individual learning trajectories. Curr Biol 34:5349–5358.e6. 10.1016/j.cub.2024.09.045 39413788 PMC11579926

[B51] Panigrahi B, et al. (2015) Dopamine is required for the neural representation and control of movement vigor. Cell 162:1418–1430. 10.1016/j.cell.2015.08.014 26359992

[B52] Park J, Phillips JW, Guo JZ, Martin KA, Hantman AW, Dudman JT (2022) Motor cortical output for skilled forelimb movement is selectively distributed across projection neuron classes. Sci Adv 8:eabj5167. 10.1126/sciadv.abj5167 35263129 PMC8906739

[B53] Parker N, Cameron C, Taliaferro J, Lee J, Choi JY, Davidson TJ, Daw ND, Witten IB (2016) Reward and choice encoding in terminals of midbrain dopamine neurons depends on striatal target. Nat Neurosci 19:845–854. 10.1038/nn.4287 27110917 PMC4882228

[B54] Ramanathan S, Hanley JJ, Deniau JM, Bolam JP (2002) Synaptic convergence of motor and somatosensory cortical afferents onto GABAergic interneurons in the rat striatum. J Neurosci 22:8158–8169. 10.1523/JNEUROSCI.22-18-08158.2002 12223570 PMC6758073

[B55] Ranieri F, Pellegrino G, Ciancio AL, Musumeci G, Noce E, Insola A, Diaz Balzani LA, Di Lazzaro V, Di Pino G (2022) Sensorimotor integration within the primary motor cortex by selective nerve fascicle stimulation. J Physiol 600:1497–1514. 10.1113/JP282259 34921406 PMC9305922

[B56] Reppert TR, Lempert KM, Glimcher PW, Shadmehr R (2015) Modulation of saccade vigor during value-based decision making. J Neurosci 35:15369–15378. 10.1523/JNEUROSCI.2621-15.2015 26586823 PMC4649007

[B57] Resulaj A, Kiani R, Wolpert DM, Shadlen MN (2009) Changes of mind in decision-making. Nature 461:263–266. 10.1038/nature08275 19693010 PMC2875179

[B58] Sachidhanandam S, Sreenivasan V, Kyriakatos A, Kremer Y, Petersen CC (2013) Membrane potential correlates of sensory perception in mouse barrel cortex. Nat Neurosci 16:1671–1677. 10.1038/nn.3532 24097038

[B59] Sanabria BD, Baskar SS, Yonk AJ, Linares-Garcia I, Abraira VE, Lee CR, Margolis DJ (2024) Cell-type specific connectivity of whisker-related sensory and motor cortical input to dorsal striatum. eNeuro 11:ENEURO.0503-23.2023. 10.1523/ENEURO.0503-23.2023 38164611 PMC10849041

[B60] Sanders JI, Hangya B, Kepecs A (2016) Signatures of statistical computation in the human sense of confidence. Neuron 90:499–506. 10.1016/j.neuron.2016.03.025 27151640 PMC5350614

[B61] Shadmehr R, et al. (2019) Movement vigor as a reflection of subjective economic utility. Trends Neurosci 42:323–336. 10.1016/j.tins.2019.02.003 30878152 PMC6486867

[B62] Stone C, Mattingley JB, Rangelov D (2022) On second thoughts: changes of mind in decision-making. Trends Cogn Sci 26:419–431. 10.1016/j.tics.2022.02.004 35279383

[B63] Tai LH, Lee A, Benavidez N, Bonci A, Wilbrecht L (2012) Transient stimulation of distinct subpopulations of striatal neurons mimics changes in action value. Nat Neurosci 15:1281–1289. 10.1038/nn.3188 22902719 PMC3951287

[B64] Takikawa Y, Kawagoe R, Itoh H, Nakahara H, Hikosaka O (2002) Modulation of saccadic eye movements by predicted reward outcome. Exp Brain Res 142:284–291. 10.1007/s00221-001-0928-1 11807582

[B65] Turner KM, Balleine BW (2024) Stimulus control of habits: evidence for both stimulus specificity and devaluation insensitivity in a dual-response task. J Exp Anal Behav 121:52–61. 10.1002/jeab.898 38100179 PMC10953355

[B66] Ursell T (2025). Frechet distance calculator. Available online at: https://www.mathworks.com/matlabcentral/fileexchange/41956-frechet-distance-calculator, MATLAB Central File Exchange (Retrieved March 12, 2025).

[B67] Wagner MJ, Savall J, Kim TH, Schnitzer MJ, Luo L (2020) Skilled reaching tasks for head-fixed mice using a robotic manipulandum. Nat Protoc 15:1237–1254. 10.1038/s41596-019-0286-8 32034393 PMC7586302

[B68] Wang AY, et al. (2013) The dorsomedial striatum encodes net expected return, critical for energizing performance vigor. Nat Neurosci 16:639–647. 10.1038/nn.3377 23584742 PMC3651899

[B69] Roth RH, et al. (2024) Thalamic integration of basal ganglia and cerebellar circuits during motor learning. bioRxiv: 2024.2010.2031.621388.

[B70] Yang L, Masmanidis SC (2020) Differential encoding of action selection by orbitofrontal and striatal population dynamics. J Neurophysiol 124:634–644. 10.1152/jn.00316.2020 32727312 PMC7500377

[B71] Yttri E, Dudman J (2016) Opponent and bidirectional control of movement velocity in the basal ganglia. Nature 533:402–406. 10.1038/nature17639 27135927 PMC4873380

