# Supplementary figures and images for: An Open-Source Joystick Platform for Investigating Forelimb Motor Control, Auditory-Motor Integration, and Value-Based Decision-Making in Head-Fixed Mice
Source: eNeuro. 2025 Apr 18;12(4):ENEURO.0038-25.2025. doi: 10.1523/ENEURO.0038-25.2025 (PMC12037168; doi:10.1523/ENEURO.0038-25.2025)

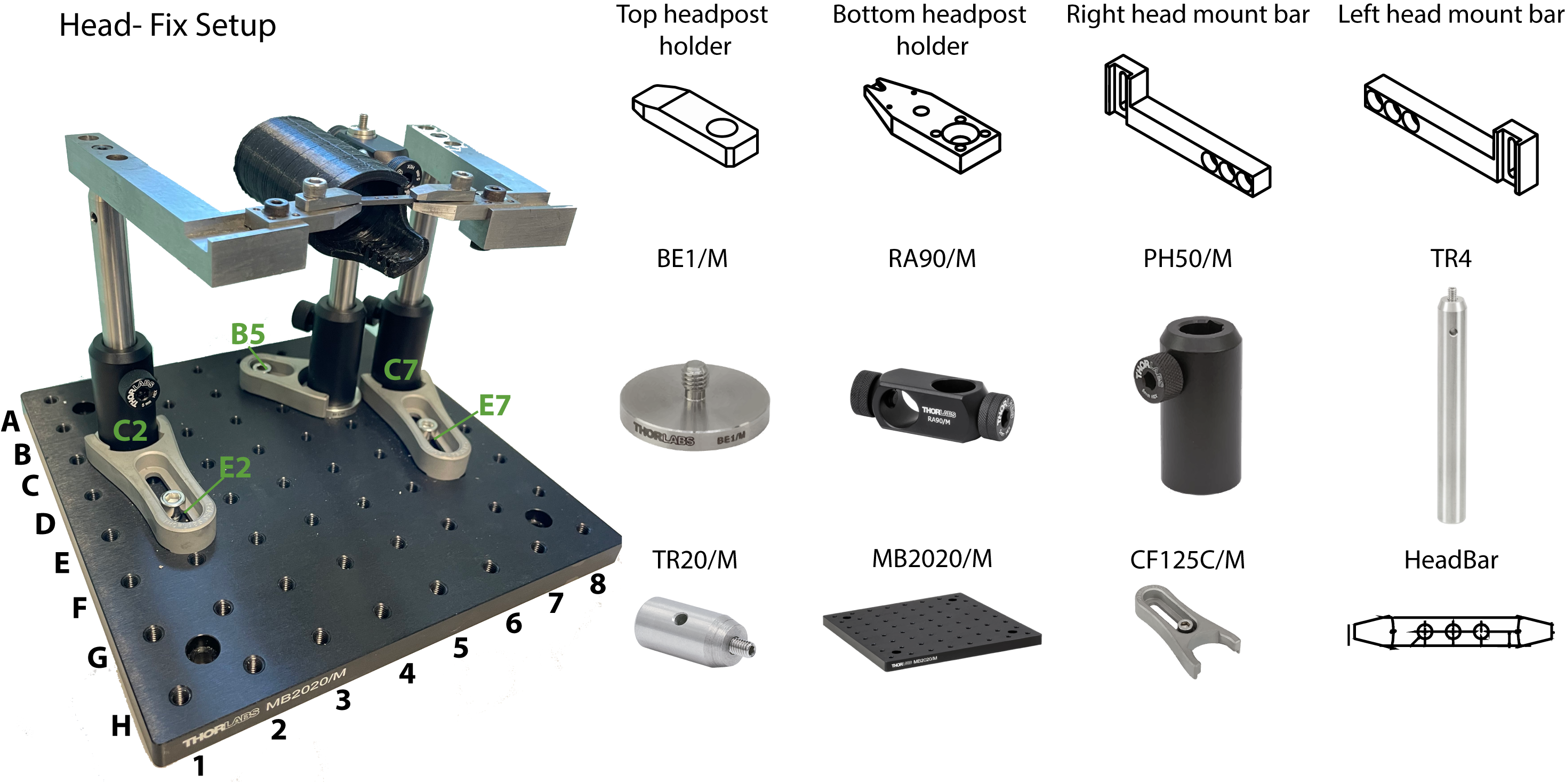

Supplement: Extended Figure 1 — Download Extended Figure 1, TIF file. [file eneuro-12-ENEURO.0038-25.2025-s004.tif]

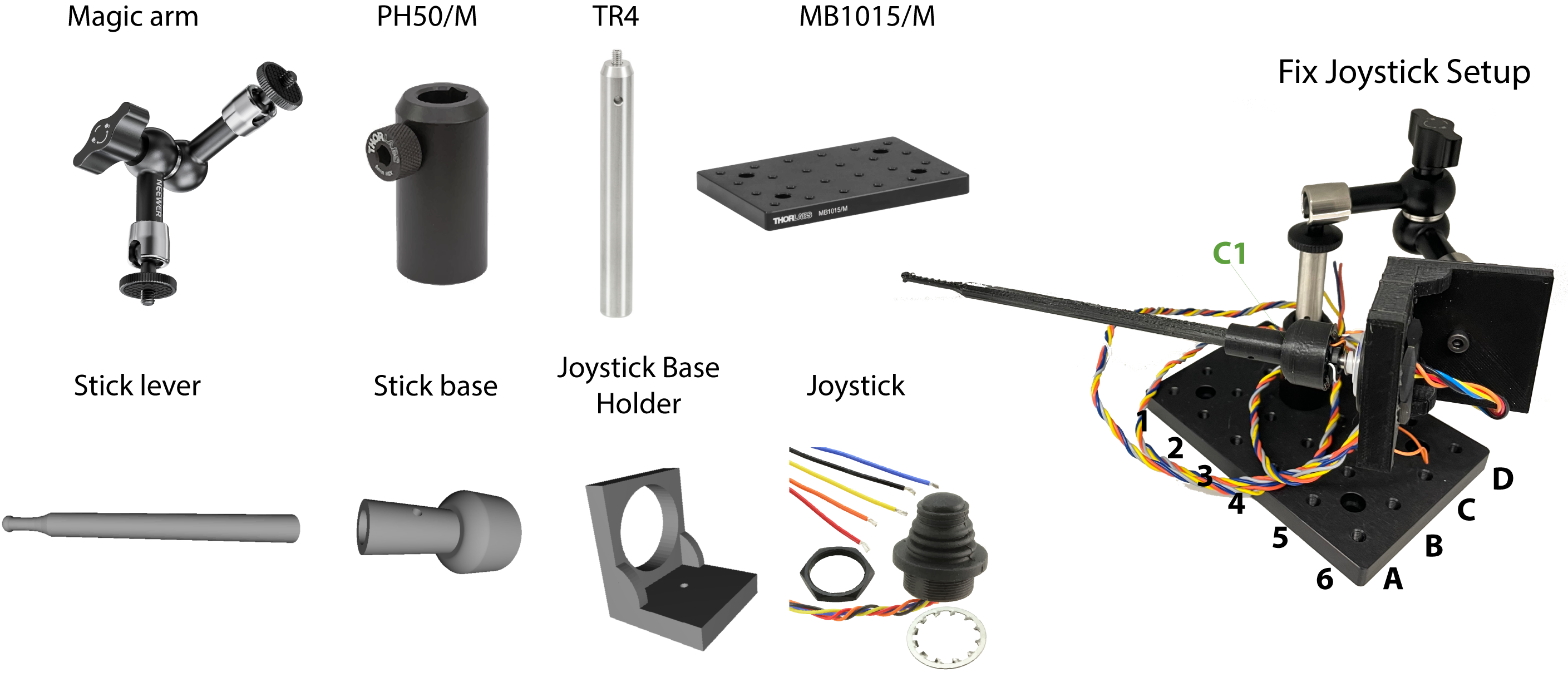

Supplement: Extended Figure 2 — Download Extended Figure 2, TIF file. [file eneuro-12-ENEURO.0038-25.2025-s005.tif]

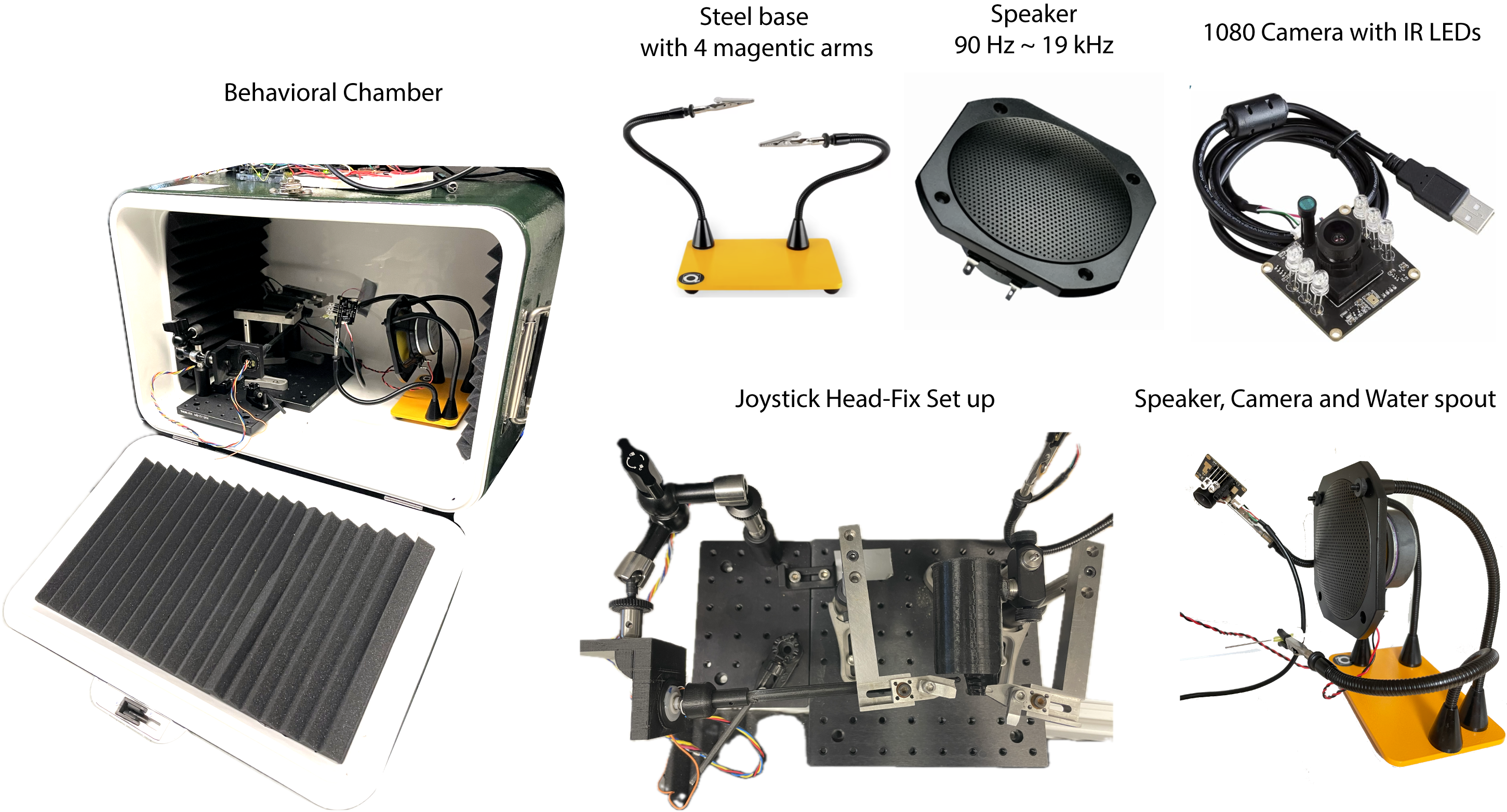

Supplement: Extended Figure 3 — Download Extended Figure 3, TIF file. [file eneuro-12-ENEURO.0038-25.2025-s006.tif]

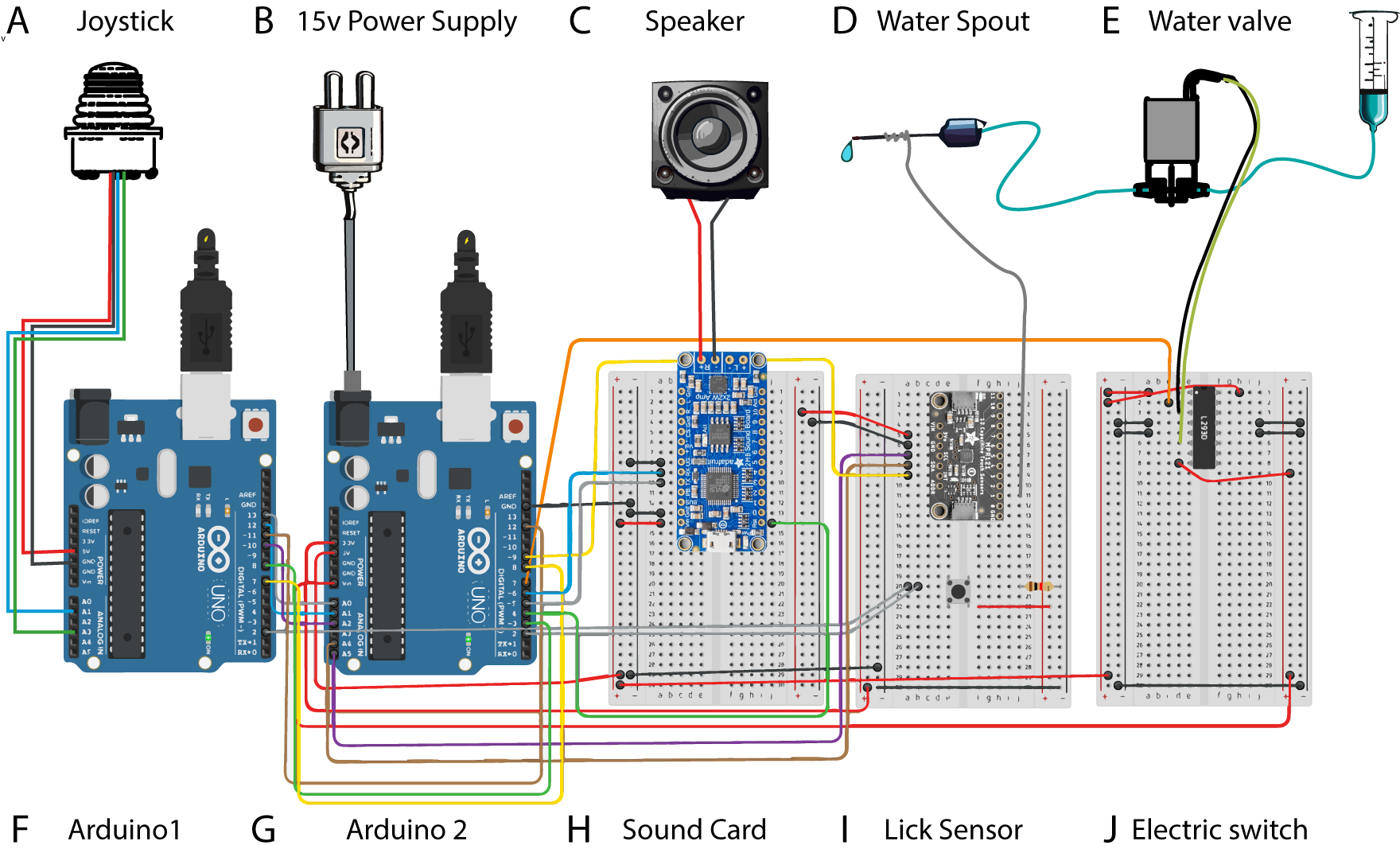

Supplement: Extended Figure 4 — Download Extended Figure 4, TIF file. [file eneuro-12-ENEURO.0038-25.2025-s007.tif]

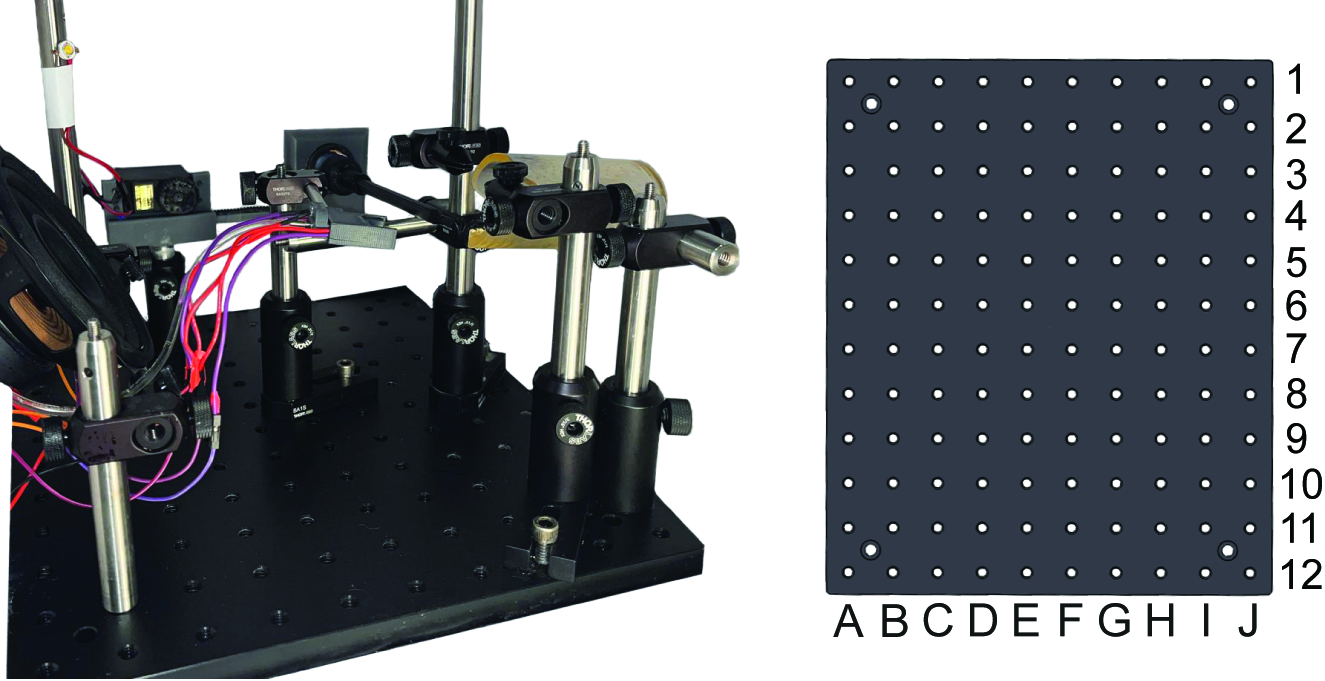

Supplement: Extended Figure 5 — Download Extended Figure 5, TIF file. [file eneuro-12-ENEURO.0038-25.2025-s008.tif]

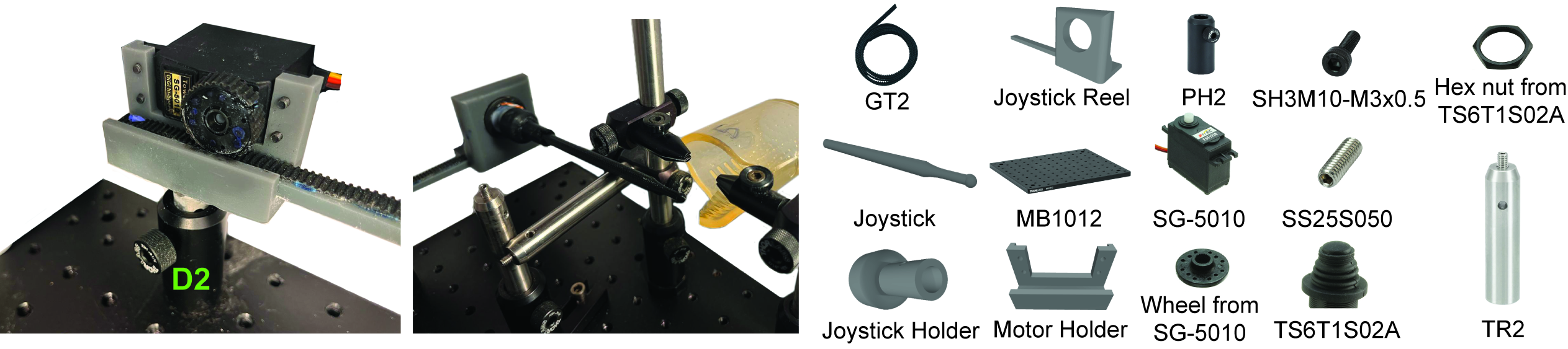

Supplement: Extended Figure 6 — Download Extended Figure 6, TIF file. [file eneuro-12-ENEURO.0038-25.2025-s009.tif]

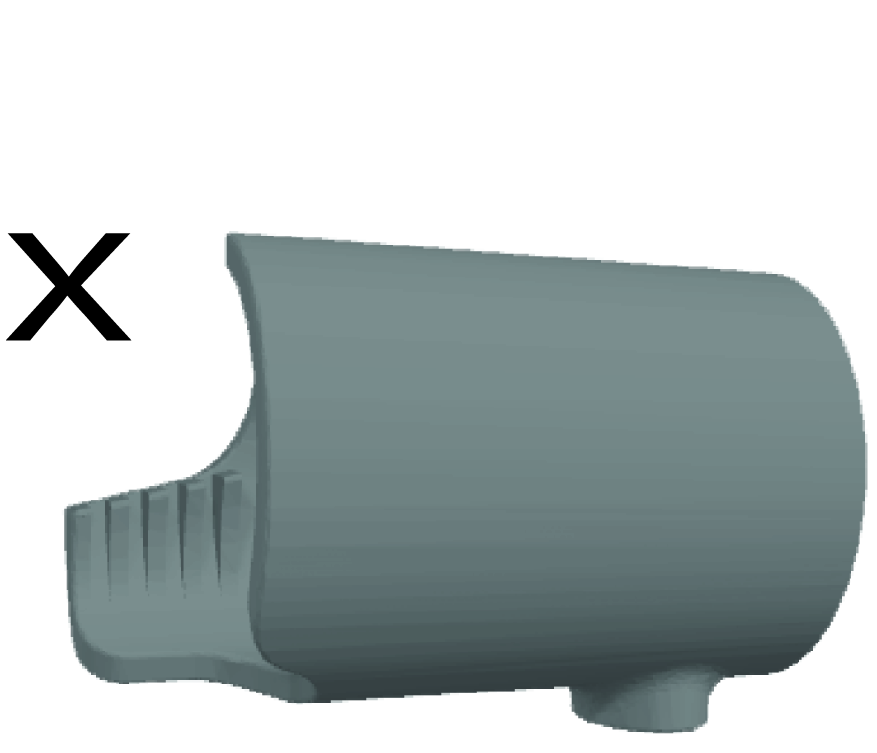

Supplement: Extended Figure 7 — Download Extended Figure 7, TIF file. [file eneuro-12-ENEURO.0038-25.2025-s010.tif]

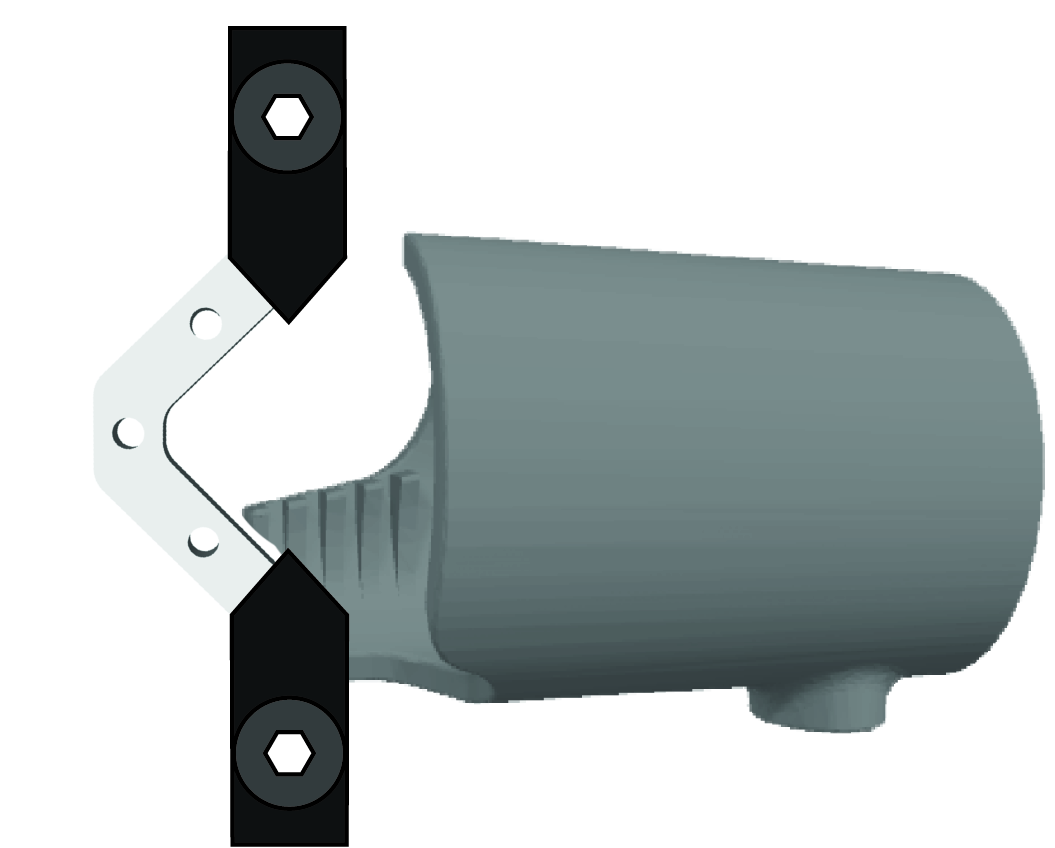

Supplement: Extended Figure 8 — Download Extended Figure 8, TIF file. [file eneuro-12-ENEURO.0038-25.2025-s011.tif]

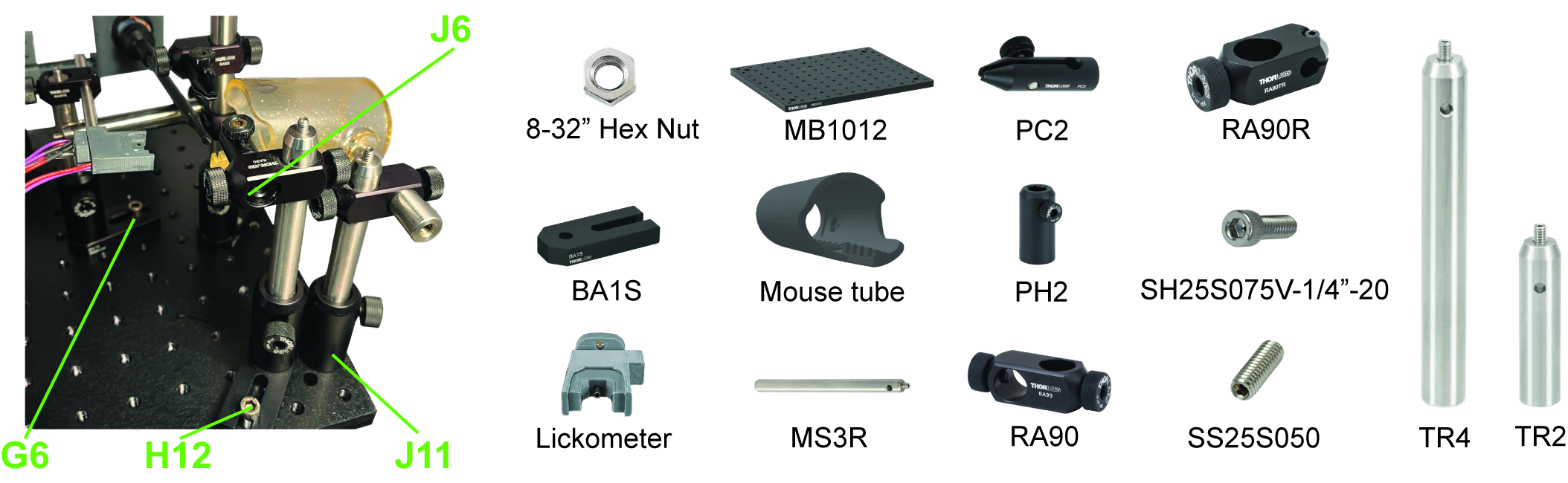

Supplement: Extended Figure 9 — Download Extended Figure 9, TIF file. [file eneuro-12-ENEURO.0038-25.2025-s012.tif]

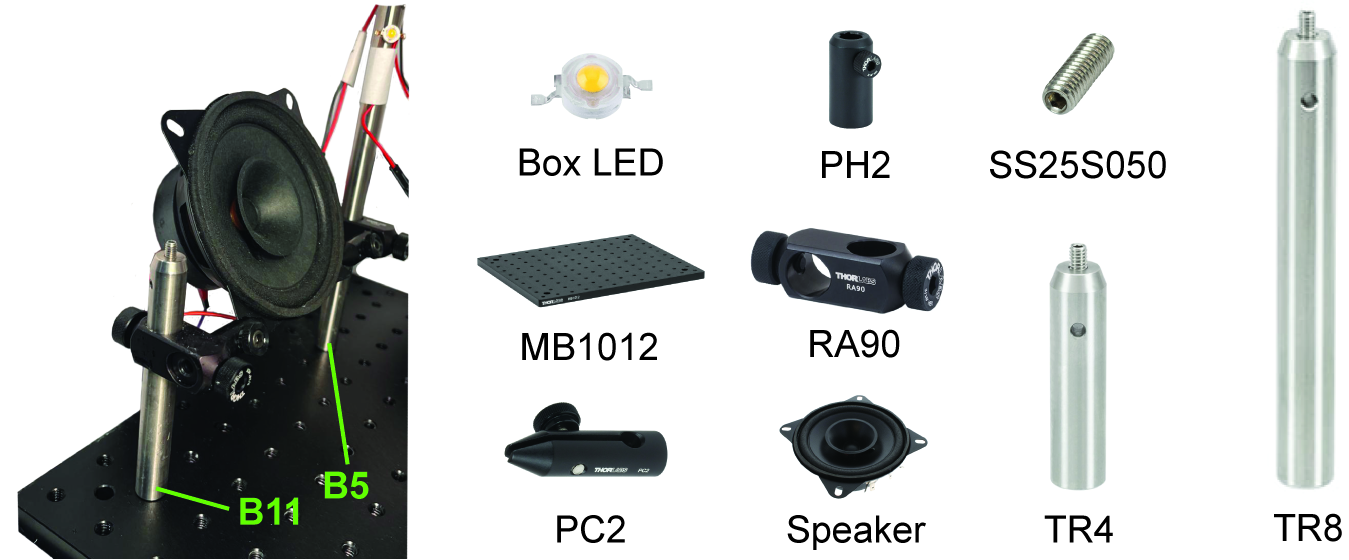

Supplement: Extended Figure 10 — Download Extended Figure 10, TIF file. [file eneuro-12-ENEURO.0038-25.2025-s013.tif]
